# The Role of Pleiotropy and Epistasis on Evolvability and Robustness in a Two-Peak Fitness Landscape

**DOI:** 10.3390/biology13121003

**Published:** 2024-12-02

**Authors:** Priyanka Mehra, Arend Hintze

**Affiliations:** 1 Department for MicroData Analytics, Dalarna University, 791 88 Falun, Sweden; pmh@du.se; 2 BEACON Center for the Study of Evolution in Action, Michigan State University, East Lansing, MI 48824, USA

**Keywords:** epistasis, pleiotropy, evolvability, robustness, survival of the flattest

## Abstract

Understanding the balance between robustness and evolvability is crucial in evolutionary dynamics. This study aims to determine how varying mutation rates and valley depths affect this interplay during adaptation. Using a two-peak fitness landscape model requiring populations to cross a fitness valley to reach a higher peak, we investigate how mutation rates and valley depths influence both evolvability—the capacity to generate beneficial mutations—and mutational robustness, which stabilizes populations at the highest peak. Our experiments reveal that at low mutation rates, populations struggle to cross fitness valleys, reducing the occurrence of pioneers. As mutation rates increase, valley crossing becomes more frequent, but organisms forming a majority at the highest peak are less common and tend to arise at intermediate mutation rates. Although pioneers reach the highest peak, they are often replaced by more mutationally robust organisms that later form a majority. This suggests that while evolvability aids in valley crossing, long-term stability at the highest peak requires greater mutational robustness. Our findings highlight that adaptations in epistasis and pleiotropy facilitate the trade-off between evolvability and robustness, providing insights into how organisms navigate complex fitness landscapes. These results can also inform the design of genetic algorithms that balance evolvability with robustness to optimize outcomes.

## Simple Summary

Understanding how organisms balance robustness against mutations with the ability to evolve is fundamental in evolutionary biology. This study uses a computational model of a two-peak fitness landscape separated by a fitness valley. We explore how different mutation rates and valley depths impact the evolutionary adaptation of organisms evolving from the lower to the higher peak. Our findings show that at low mutation rates, populations rarely cross the valley to reach the higher fitness peak. As mutation rates increase, crossing becomes more frequent, but those who find the peak first (pioneers) struggle to form a stable majority at the newfound peak. Instead, highly evolvable pioneers become increasingly more often outcompeted by more mutationally robust competitors. This suggests that while high evolvability facilitates crossing fitness valleys, long-term stability at the highest peak relies on greater mutational robustness. Additionally, our results highlight how genetic interactions like epistasis and pleiotropy mediate the trade-off between evolvability and robustness. These insights not only enhance our understanding of evolutionary dynamics but also inform the design of genetic algorithms by emphasizing the need to balance adaptability and stability for optimal performance.

## 1. Introduction

Robustness and evolvability are defining properties of biological systems, representing two seemingly contradictory yet fundamentally interconnected facets of evolution [1]. Robustness refers to the ability of an organism to maintain its functions despite genetic mutations and environmental changes [2]. Evolvability, on the other hand, is the capacity of a population to generate phenotypic variation, which can be acted upon by natural selection [3]. Robustness minimizes the impact of genetic mutations, maintaining stability within populations. This stability, however, might limit phenotypic variation, which is crucial for evolvability. In other words, if an organism is too robust, it may not generate enough variation for natural selection to act upon, potentially hindering adaptive evolution. Due to this, robustness and evolvability appear to be at odds with each other. However, researchers have explored how robustness and evolvability can not only coexist but also support each other [4,5,6].

Studies suggest that robust systems can accumulate hidden genetic variations. These hidden variations, or cryptic genetic variations, are mutations that do not affect the organism under current conditions but can become beneficial if the environment changes [5]. This concept means that while an organism is robust, it can safely store these hidden variations without any negative impact. When the environment changes or the organism’s robustness is somehow reduced, these previously hidden variations can suddenly become important. They provide a new source of genetic diversity that can help the organism to adapt quickly to new conditions. In this way, robustness helps by preparing the organism for future changes, enhancing its evolvability [5].

Other researchers have demonstrated that robustness can facilitate adaptation. They showed that robust populations can explore a wider range of genetic mutations without suffering immediate detrimental effects. This exploration allows these populations to discover beneficial mutations that might not have been accessible otherwise. When the environment changes, these beneficial mutations can quickly become advantageous, enabling rapid adaptation [6]. Additionally, robustness can help populations move across the adaptive landscape, which is a metaphorical representation of how different combinations of traits affect an organism’s fitness. Sometimes, to reach a higher peak of fitness, a population must first cross a valley of lower fitness. Robustness helps by stabilizing intermediate forms or traits, allowing the population to survive while it transitions through these less optimal states [7]. This stabilization makes it easier for the population to eventually reach higher fitness peaks, showing that robustness can facilitate significant evolutionary changes [7].

Building on these concepts of robustness and evolvability, previous research has sought to understand how these properties interact within evolutionary dynamics. Epistasis (ϵ), which involves interactions between different genetic loci, and pleiotropy (π), where a single gene affects multiple traits, are crucial factors in this context.

Pleiotropy refers to a genetic phenomenon where a single gene influences multiple phenotypic traits [8]. This means that a mutation in a pleiotropic gene can have cascading effects on various traits, potentially affecting an organism’s fitness in multiple ways. Epistasis, on the other hand, describes interactions between different genetic loci, where the effect of one gene is modified by one or several other genes [9]. Epistatic interactions can lead to non-additive effects on phenotypes, creating complex genetic architectures that influence how organisms respond to mutations.

Both pleiotropy and epistasis are fundamental in shaping evolutionary trajectories because they determine how genetic variations translate into phenotypic changes [10]. Pleiotropy can constrain or facilitate evolution by linking the fate of multiple traits, making it more challenging for selection to optimize each trait independently [8]. Epistasis contributes to the ruggedness of fitness landscapes by creating peaks and valleys resulting from gene interactions, thereby affecting the accessibility of adaptive pathways [11].

However, these genetic mechanisms play a dual role during evolutionary adaptation: they influence how organisms respond to mutations while they themselves also adapt. These interactions can either buffer organisms from harmful mutations, contributing to robustness, or enhance variability, increasing evolvability. Over successive generations, these genetic mechanisms guide the evolutionary path of populations, determining whether they maintain stability or generate beneficial variations in response to environmental pressures. The impact of ϵ and π on robustness and evolvability has been widely debated in the literature.

Increased levels of ϵ can either enhance or diminish an organism’s robustness. For instance, some studies suggest that high ϵ can lead to complex gene interactions that buffer against the effects of mutations, thereby enhancing robustness [7]. However, in some contexts, high ϵ might also lead to greater mutational load, reducing robustness and making populations more susceptible to deleterious mutations [9]. Similarly, π can have dual effects on evolvability. On one hand, π can constrain evolution by limiting the ability of organisms to adapt independently across different traits [8]. On the other hand, pleiotropic genes can also generate novel trait combinations, potentially increasing evolvability by providing a broader range of phenotypic variation for natural selection to act upon [10].

Several studies have highlighted the challenges in resolving the debate on how ϵ and π influence evolutionary outcomes [8,9,10,12,13]. The complexity arises partly due to the difficulty of conducting controlled evolutionary experiments with natural organisms, where the intricate networks of genetic interactions are difficult to manipulate and observe directly [13]. Additionally, experiments with natural organisms can be time-consuming, costly, and limited by ethical considerations. Computational (in silico) models offer significant advantages in this context, providing lower cost, faster results, and the ability to explore a wide range of parameters and conditions systematically [14,15]. These models allow for precise control over variables like mutation rates and fitness landscapes, facilitating the study of complex evolutionary dynamics that would be impractical to investigate experimentally. Recent advancements in computational modeling, such as our extension of the classic NK fitness landscape model, have provided new ways to independently analyze the effects of epistasis, pleiotropy, and ruggedness on evolutionary adaptation [16]. In our model, we utilize indirect encoding through a simple matrix that maps genetic sites to phenotypic traits, providing a straightforward method to study these interactions. With the ability to analyze ϵ and π, we can investigate how these factors impact robustness and evolvability in a controlled and efficient manner.

A key framework for this discussion is the “Survival of the Flattest” (SoF) phenomenon, which highlights a paradox where, under conditions of high mutation rates, populations residing on flatter, less optimal fitness peaks can outperform those on higher, steeper peaks due to their greater resistance to mutations [17]. This insight suggests that, in mutationally stressful environments, populations may prioritize stability over peak fitness to ensure long-term survival. In our previous work [18], we investigated how reducing epistasis and pleiotropy could help avoid the SoF tragedy. Our findings challenge the expectation that populations would drift toward flatter peaks under high mutation rates [17,19]. Instead, we demonstrated that by minimizing the complexity of these genetic interactions (reducing ϵ and π), organisms can maintain their fitness advantage even in highly mutational environments.

However, this previous work primarily focused on populations already at a higher fitness peak, showing that reducing ϵ and π allows them to maintain higher mutational robustness and stay at that peak for longer periods. The critical question of how populations transition from a lower fitness peak to a higher one and stay there in a two-peak landscape remained unanswered. This research seeks to address that gap by investigating the dynamics of an organism’s movement from lower to higher fitness peaks, providing new insights into the interplay between evolvability and robustness. However, this question creates an enigma: on the one hand, evolvable organisms should be able to cross the valley better [20,21], but on the other hand, we already know that to stay at the higher peak, organisms need higher mutational robustness [18].

Some researchers argue that robustness and evolvability are opposing forces [1,22,23]. Others, however, propose that robustness and evolvability can be independent traits [2,5,24]. Robustness and evolvability can coexist within an organism, where robustness ensures survival in stable environments, while evolvability becomes advantageous when environments change, allowing for the generation of beneficial mutations. This perspective sees both as being able to coexist without directly affecting each other. A third view posits that robustness and evolvability are orthogonal, meaning that they operate in different dimensions and can influence each other under certain conditions without being directly opposed or independent [6,25]. The relationship between robustness and evolvability is complex and multi-faceted, with different researchers supporting different ideas.

In this study, we aim to investigate the dynamics of population movement from lower to higher fitness peaks in a two-peak fitness landscape, focusing on the roles of evolvability and mutational robustness. Specifically, we explore how varying μ (ranging from 0.0001 to 0.05), valley depth (0.1, 0.3, 0.5, and 0.7), and the height of secondary peaks (0.5 and 0.7) influence both the ability of populations to cross fitness valleys—requiring high evolvability—and the capacity to remain stable at the highest fitness peak—requiring mutational robustness.

Our computational model allows us to test how these factors affect the evolutionary trajectories of populations as they move across the adaptive landscape. In particular, we examine how populations transition from pioneers—organisms that reach the highest peak first—to those capable of forming a majority and maintaining their position at the highest peak. We also analyze how mutational effects change over time, as populations adapt their epistasis (ϵ) and pleiotropy (π) to either enhance evolvability or increase robustness, depending on the mutation rate and environmental conditions. Through our computational experiments, we aim to shed light on this complex interplay and provide insights into the conditions that promote successful adaptation.

## 2. Materials and Methods

### 2.1. Indirect Encoding

**Figure 1:**
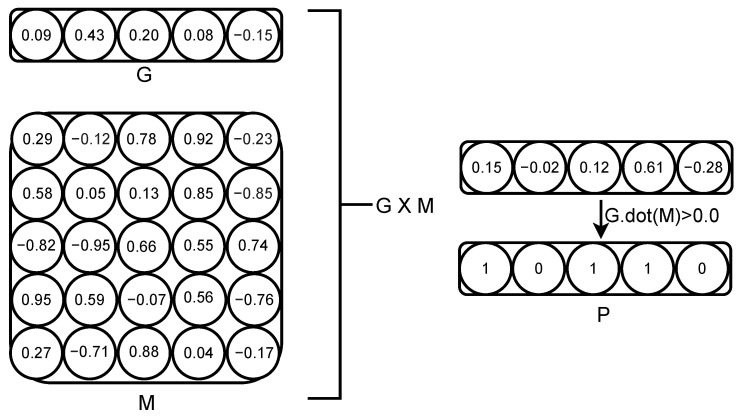
Each organism’s genotype consists of *N* genes, where each gene is associated with a specific value and a corresponding vector of length *N*. These gene vectors collectively form the interaction matrix *M*, while the gene values themselves create the vector *G*. The phenotype vector is derived from the dot product of *G* and *M*. If the result of the dot product for a given trait is greater than 0, the corresponding phenotypic trait is set to 1; otherwise, it is set to 0. This process results in a binary phenotype vector. Mutations can alter all values within the genotype, meaning the interaction between *G* and *M* can indirectly influence the phenotype. In contrast, with direct encoding, *M* would be a fixed identity matrix, leading to direct and immutable gene-to-trait mappings.

The indirect encoding approach differentiates between an organism’s genotype and phenotype. The genotype is composed of *N* genes, each assigned an expression value that can range from −1.0 to 1.0, similar to how catalytic activity might be quantified. These genes can interact with any of the *N* genes, including self-interactions, with interaction strengths also ranging from −1.0 to 1.0 (as illustrated in Figure 1). The phenotype is derived by computing the dot product of the expression values *G* with the interaction weights *M*. The resulting vector, which represents the phenotype, is then discretized: positive values are converted to 1, while all other values are set to 0. Importantly, in this model, the ability of genes to interact is not fixed; it can evolve over time by modifying the expression values *G* and the interaction weights in matrix *M*, independently of *K*.

### 2.2. Epistasis and Pleiotropy

In our extended model [16], we separate the genetic interactions that determine traits, allowing these interactions to evolve independently. This necessitates an independent measurement of epistasis (ϵ) and pleiotropy (π).

**Figure 2:**
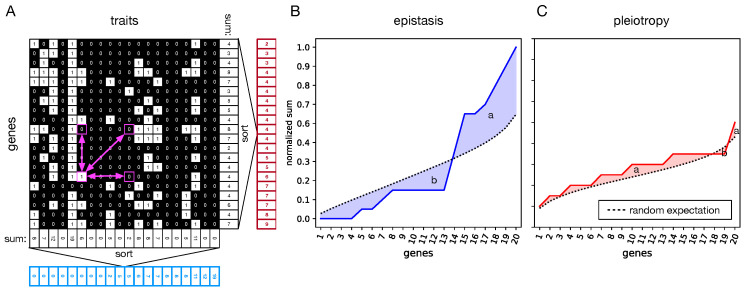
Illustration how epistasis ϵ and pleiotropy π are calculated as the difference between the expected and measured interactions. Panel (**A**) shows an organism’s interaction matrix (IM); the rows correspond to the N=20 genes, and the columns correspond to the N=20 traits. A 0 indicates no interaction between gene and trait, while a 1 identifies that a gene influences a trait. The sum of each row (pleiotropy) and the sum of each column (epistasis) are computed, and the resulting vectors are sorted in ascending order. Panel (**B**) shows the resulting (normalized) epistasis vector in blue and the difference between the random expectation (dashed black line) as a blue surface. The part above the expectation is labeled “a”, while the surface below the expectation is labeled “b”. Panel (**C**) is the same as panel (**B**) but it shows the pleiotropy results (in red) and the random expectation for pleiotropy (dashed black line). The total difference between measured and random expectation is then calculated as a−b. Most of the time, the observed values (solid blue or red line) do not cross the expected line (black line) but remain consistently on one side. However, this method accounts for cases where the values fluctuate around the expectation, ensuring accurate measurement of the differences. Observe that a change in the interaction matrix, such as purple arrows in panel A indicating a swap of interactions, can affect pleiotropy and epistasis differently. For example, a horizontal swap can alter epistasis, a vertical swap affects pleiotropy, and a diagonal affects both. The addition or removal of interactions at a single location would affect both pleiotropy and epistasis at the same time.

We calculate an interaction matrix (IM) that indicates how each gene influences individual traits. Given that each organism has *N* genes and *N* traits, the IM is a square matrix with dimensions N×N [16]. To assess whether a gene affects a particular trait, we alter its expression value $G_{i}$ between −1.0 and 1.0. For each of these values, the phenotype (P=G×M) is constructed, and we determine whether the change in gene *i* (row) impacts trait *j* (column). If it does, the interaction matrix $IM_{i,j}$ is assigned a value of 1; if not, it is assigned a value of 0. Each row in this matrix, thus, represents a gene, while each column represents a trait. Consequently, every 1 in a row indicates that gene *i* influences trait *j*. The sum of a row is thus the number of traits affected by that gene, and so the sum of a row quantifies the pleiotropy (π) of the genes *i*. Conversely, each 1 in a column identifies that gene *i* contributes to trait *j*. The sum of a column is, therefore, the degree of epistasis (ϵ) or how many genes contribute to that trait (see Figure 2A for an illustration). The mapping of genes to traits is governed by the mapping matrix (*M*), which evolves over time. Since mutations occur randomly, direct comparisons of the resulting π and ϵ vectors between two organisms are not straightforward. To address this, we sort these vectors in ascending order [16], which allows us to analyze the overall distribution across the entire organism rather than focusing on the specific ϵ or π of individual genes.

We used a method to measure how ϵ and π differ from what is expected randomly (represented by the black line; see Figure 2B,C). The random expectation is calculated using 1.000 mapping matrices with values randomly drawn from a uniform distribution ranging from −1 to 1. This approach yields a negative value if ϵ and π are lower than expected by chance, and a positive value if they are higher [16].

To quantify the difference between the observed and expected ϵ or π, we calculate the area between the two curves. We independently calculate the total area above the random expectation and the area below it, denoting them as *a* and *b*, respectively. The difference between these areas gives us the distance Δ, representing the discrepancy between the observed values and the random expectation.

### 2.3. Mutations

In our model, mutations occur during reproduction. Each time an organism is selected to produce an offspring for the next generation, each part of its genome has an independent chance to mutate. The mutation rate (μ) is a per-site (per locus) mutation rate, meaning that every genome element has a probability μ of undergoing mutation during reproduction.

The genome comprises two components: the expression vector *G* and the interaction matrix *M*. The expression vector *G* contains *N* elements, and the interaction matrix *M* contains N×N elements corresponding to the interactions between genes. Therefore, the genome has a total of N+N×N=N(N+1) mutable sites.

At initialization, the values in *G* and *M* are drawn from a uniform random distribution between −1.0 and 1.0. During mutation, if a site (an element of *G* or *M*) is selected to mutate, its value is replaced with a new value randomly drawn from the same uniform distribution between −1.0 and 1.0. This means mutations completely replace the parental value with a new value from the allowed range rather than slightly changing the existing value. This approach ensures that all mutated values remain within the valid range of −1.0 to 1.0, maintaining consistency in the genome’s representation.

### 2.4. Two-Peak Model

**Figure 3:**
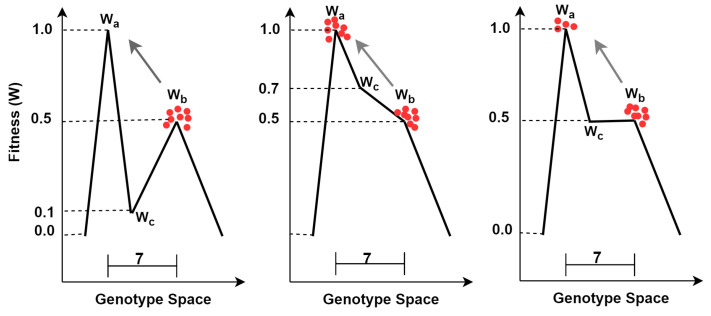
Illustration of the survival of the flattest phenomenon. When comparing the two peaks of different heights, one would expect the population to sit at the highest (primary) and not a lower adjacent one (secondary). However, if the mutation rate and the mutation effect size (horizontal arrows) are strong enough, populations (red dots) converge on the lower one. The y-axis illustrates the fitness of all organisms, and the x-axis depicts the mutational distance between genotypes. The scale bars show the mutational distance between peaks (7) or the width of the primary (2) and secondary peak (5).

The computational model is designed to investigate how populations adapt within a two-peak fitness landscape, specifically to assess the impact of evolvability and robustness on their ability to transition between fitness peaks and maintain long-term stability at the higher peak. In this landscape (see Figure 3), the traits are represented by a binary vector of length N=20. The landscape includes two distinct fitness peaks, with the primary peak assigned a fitness value of $W_{a}=1.0$, and the secondary peak assigned a variable fitness value $W_{b}$ depending on the experimental conditions ($W_{b}=0.5$ or $W_{b}=0.7$). These peaks are separated by a Hamming distance of 7, meaning that the phenotypes corresponding to these peaks differ at exactly 7 positions. The fitness values for the mutational neighborhood surrounding the secondary peak decrease linearly with increasing Hamming distance from the peak, similar to previous models.

The valley between these peaks is characterized by a fitness value $W_{c}$, which varies between 0.1, 0.3, 0.5, and 0.7, representing the depth of the fitness valley. The fitness valley is a single genotype/phenotype exactly two mutations away from the higher peak, and five mutations away from the lower peak. A single mutational path from the highest peak, over the valley, to the lower peak is identified, and fitnesses along this path are linearly interpolated. A key difference in this model compared to earlier works [17,26,27] is the use of indirect encoding. In this model, while the phenotype’s fitness is directly determined by the landscape, the genotype–phenotype mapping introduces additional complexity. Two random genotypes are generated until a pair is found whose phenotypes have a Hamming distance of 7. These phenotypes are then used to define the fitness peaks and their respective mutational neighborhoods.

In the experiments, the entire population starts at the secondary peak. This setup is achieved by first generating a random genotype that defines the phenotype corresponding to the secondary peak. Then, a second genotype is found whose phenotype differs by exactly 7 mutations, corresponding to the primary peak. This ensures that the population is initially located at the lower-fitness secondary peak, allowing for the study of how mutation rates (μ) and other parameters influence the population’s ability to transition to and maintain its position at the higher-fitness primary peak over time.

Organisms evolve over 10,000 generations under specified mutation rates (from low (0.0001) to high (0.05)). The experiment tracks whether the population manages to migrate from the secondary peak to the primary peak and whether they can maintain their position at this higher peak over time. A peak is considered vacated if no individuals remain within its mutational neighborhood. This setup allows for an investigation into how populations navigate the two-peak fitness landscape, with a focus on the effects of indirect encoding, evolvability, and robustness on their ability to achieve and maintain high fitness in the long term.

### 2.5. Mutational Robustness and Evolvability

Mutational robustness can be measured differently [4]. Here, we use the mean effect that mutations have on the phenotype to quantify mutational robustness. Technically, the larger the mean mutational effect on the phenotype, the lower the mutational robustness. In turn, the easier mutation can be spawned to find new places in the landscape. To assess the mean effect on the phenotype, we measure the mean Hamming distance of mutants ($\bar{HD}$) compared to the organism in question when applying mutations. To that end, the organism in question produces 10,000 offspring with a point mutation rate of 1%—observe that these mutations are applied to the genotype, specifically the expression levels and interaction weights. For those 10,000 mutants, the phenotype and, with it, the Hamming distances are computed and averaged.

### 2.6. Artificially Increasing Epistasis, Pleiotropy, and Mutational Robustness

When correlating the mean epistatic or pleiotropic effects to $\bar{HD}$, randomly generated organisms show variations that are too slight in ϵ or π. Similarly, when trying to compete organisms with high mutational robustness against those with less of it, randomly generated organisms do not differ enough. To that end, we applied a hill-climber that can increase or decrease epistasis and pleiotropy and, thus, mutational robustness.

Assuming that we want to increase ϵ and π for a given random start organism, a mutant is created using a mutation rate of μ=0.01. Both Δϵ and Δπ are calculated, and if the mutant has a higher Δϵ+Δπ than its ancestor, it is chosen to become the new ancestor. If not, the mutant is discarded. This process of always picking the organism with a higher Δϵ+Δπ is repeated 1000 times, resulting in about 50 organisms with increasing epistasis and pleiotropy.

The same process can be used to reduce Δϵ and Δπ by picking the mutant when it has a lower Δϵ+Δπ [18].

### 2.7. Details of the Computational Model

The code for the computational model can be found here https://osf.io/szr5m/ last update 11 November 2024. Unless stated otherwise, we used populations of 100 organisms that evolved for 10,000 generations, given the different environmental conditions, such as the height of the secondary peak, the height of the valley, and the mutation rate. Organisms were generated at random, drawing each value of the expression vector (*G*) and the interaction weights (*M*) from a uniform random distribution between −1.0 and 1.0. The selection was performed using a roulette wheel selection method, replacing the entire population at every generation proportionally to their respective fitness.

Populations were initialized with 100 identical organisms. At every generation, the entire population was replaced with a new one based on the fitness of the individuals by employing a roulette wheel selection, a stochastic selection method where the probability of an individual being chosen for reproduction is proportional to its fitness. This approach ensures that fitter individuals have a higher likelihood of passing their traits to the next generation. Additionally, the replacement process was asexual and thus without recombination or horizontal gene transfer. The population size of 100 was kept constant throughout the experiment.

All parameters of the computational model and how they were varied can be found in Table 1.

## 3. Results

In this section, we present the outcomes of our computational experiments designed to explore the roles of robustness and evolvability in the two-peak fitness landscape. We investigated how varying levels of epistasis (ϵ) and pleiotropy (π) influence the ability of populations to transition from a lower fitness peak to the highest one and maintain their position at the highest peak. We further show how ϵ and π change in order to facilitate evolutionary adaptation and their effect on evolvability and mutational robustness.

We found conflicting hypotheses regarding the relationship between robustness and evolvability in the literature. One view suggests that they are opposing forces, with robustness limiting the phenotypic variation needed for adaptation. In this view, organisms can be either mutationally robust or evolvable [1,7]. Another perspective proposes that robustness and evolvability can coexist independently, where robustness maintains stability, and evolvability drives adaptation during environmental change [5,28]. A third hypothesis posits that they are orthogonal, operating separately but influencing each other under certain conditions, with robustness potentially facilitating evolvability by accumulating cryptic genetic variation [29]. These differing perspectives highlight the complex interplay between these two traits. Given these conflicting views, we use our computational model first to confirm or reject previous expectations and then specifically test the remaining hypotheses.

From the survival of the flattest phenomenon [18], we already know that a population cannot stay at the highest fitness peak if the mutation rate is too high. If the mutation rate is somewhat lower, only organisms that reduced their ϵ or π manage to stay at the highest peak. When the mutation rate is even lower, organisms, regardless of a change in ϵ or π, can remain at the highest peak. In this two-peak model, where organisms need to cross a valley, evolutionary adaptation faces two problems: First, crossing the valley, for which one presumably needs high evolvability, and second, mutational robustness to stay at the highest peak. Depending on the relationship between mutational robustness and evolvability, these two problems might antagonize each other, be unrelated, or have a complex interaction. Besides ϵ and π, which modulate mutational robustness and evolvability, the mutation rate (μ) plays a critical role. If too low, valley crossing is impaired, while at a high level, drives it. The opposite is true for staying at the highest fitness peak. Consequently, here, we first test at what mutation rates (from low (0.0001) to high (0.05)) populations manage to evolve and remain at the highest peak. Observe that a mutation rate of 0.01 is already considered very high [30].

### 3.1. Evolvable Organisms Struggle to Form a Stable Population at High Mutation Rates

**Figure 4:**
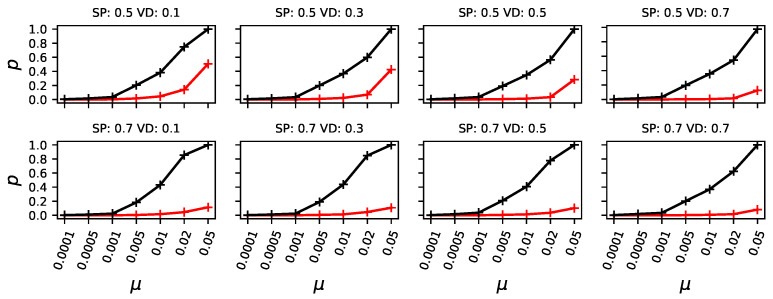
Each panel shows the likelihood (*p*) to either find the highest peak (black) or to later form a majority at the higher peak (red), given the mutation rate (μ x-axis), the height of the secondary peak (SP), top row for SP=0.5 and bottom row SP=0.7, and the fitness at the deepest point of the valley (valley depth (VD), ranging from 0.1 to 0.7 from left to right).

Given the mutation rate ranging from 0.0001 to 0.05, with two different heights of the secondary (starting) peak (0.5 and 0.7) and four differently deep valleys to cross (0.1, 0.3, 0.5, and 0.7), we conduct 10 000 independent evolutionary experiments for each combination of experimental conditions. We differentiate between pioneers, organisms that reach the highest peak first having the exact same phenotype as the highest peak, and organisms capable of forming a majority (more than 50% of the population) at the highest peak. When considering a majority, be it at the highest or the second highest peak, we consider all organisms, the ones at the peak, and all in the mutational neighborhood that belongs to each peak (all non-zero fitness phenotypes) to be a part of the population forming a majority. We found that at low mutation rates, the likelihood of crossing the valley and thus finding pioneers is seriously diminished, and only at higher mutation rates is the highest peak reliably discovered (see Figure 4, black like). Similarly, organisms forming a majority is even less likely than finding pioneers but increase as the mutation rate rises (see Figure 4, red line).

However, the chance of forming a majority at the highest peak depends not only on the mutation rate but also on the height of the secondary peak. Specifically, the higher the secondary peak, the lower the likelihood that a majority forms at the primary peak (compare Figure 4: top row with secondary peak height 0.5, and bottom row with secondary peak height 0.7). This is because a higher secondary peak reduces the fitness advantage of organisms at the primary peak, leading to increased competition and consequently making it less likely for them to form a majority.

We also find that the chance of forming a majority at the primary peak decreases as the depth of the adjacent valley decreases (i.e., as the valley becomes higher) (see Figure 4). We conjecture that when the valley is less deep, organisms from the secondary peak can more easily invade the primary peak and compete with its population (Figure 3 illustrates this phenomenon).

**Figure 5:**
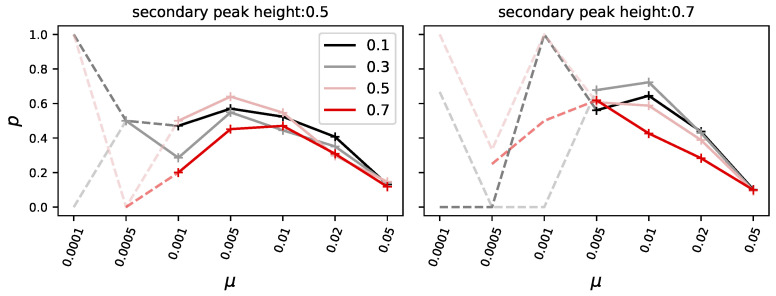
The likelihood that a descendant of the first organism to reach the highest peak is on the line of descent of those organisms who form the majority at the highest peak later. The y-axis shows a probability for the first pioneers to form a majority later. The color indicates the different fitnesses of the lowest peak in the valley (valley depth; see the color code in the legend). The left panel shows results for the secondary peak to have a height of 0.5, the right panel for 0.7. Experiments that resulted in five or fewer organisms (out of 10,000 replicates) reaching the higher fitness peak were shown as dashed lines in the same color as experiments with sufficient data.

While pioneers may reach the highest peak, they often struggle to form a majority there as the mutation rate increases. We find that the populations that eventually form a majority at the highest peak are frequently not descendants of the organism that first discovered the peak (see Figure 5). In other words, an organism can find the highest peak but fail to establish a majority despite its competitive advantage. This is notable because the organism at the highest peak has a fitness of 1.0, while all other organisms have a maximum fitness equal to that of the secondary peak (here, 0.5 or 0.7), or, in cases where the valley is higher than the secondary peak, at most 0.7.

The probability that a pioneer will form a majority at the highest peak—as opposed to being replaced by another organism that is not its descendant and thus originated from the rest of the population, likely from the secondary peak—decreases as the mutation rate increases (see Figure 5), regardless of the depth of the valley. However, the height of the secondary peak influences the likelihood that a pioneer will also become the one forming the first majority. The higher the secondary peak, the more likely the pioneer will form a majority at the highest peak. This seems reasonable, as it becomes harder for organisms on the secondary peak to cross the valley as the relative depth to overcome becomes larger (the difference between the secondary peak height and the valley becomes higher when the peak is higher). At the same time, the height of the valley has no significant impact on the chance of pioneers to form a majority at the highest peak (see Figure 5).

### 3.2. Changes to Evolvability and Mutational Robustness over the Course of Evolution

As we saw, the likelihood of crossing the fitness valley depends on the mutation rate μ, the depth of the valley, and the height of the secondary (lower) peak. Further, the pioneers find the highest peak but struggle to inhabit it, and are increasingly more often replaced by the group that forms a majority later as the mutation rate increases. This suggests that pioneers, while evolvable as they find the highest peak fast, might lack the necessary mutational robustness to remain, while those who ultimately form a majority and possibly remain at the highest peak might not share the same evolvability but possess higher mutational robustness.

**Figure 6:**
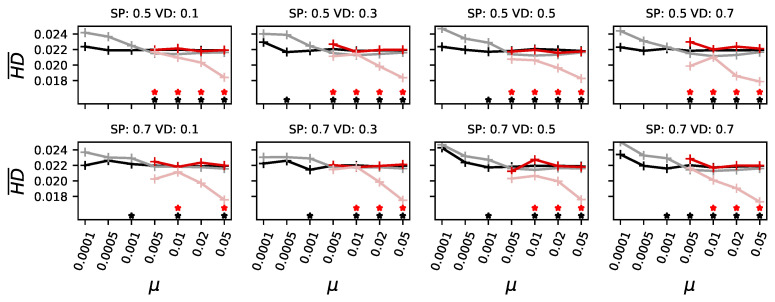
Evolvability measured as the mean Hamming distance ($\bar{HD}$) mutations caused for different organisms and different experimental conditions. In black, the ancestors of the pioneers who find the highest peak first; in gray, the adapted organisms when they reach the highest peak. In dark red, the ancestors of those organisms who will ultimately form a majority at the highest peak; the later ones in pale red. When any category had less than 0.1% data, data points were omitted. Stars indicate a significant (*p*-value of a Kolmogorov–Smirnov test less than 0.01) difference between ancestors and adapted organisms for pioneers in black, and for those forming a majority in red. Each panel shows the mutation rate (μ) on the x-axis. Panels also vary the height of the secondary peak (SP, top row for SP=0.5 and bottom row SP=0.7, and the fitness at the deepest point of the valley (valley depth (VD), ranging from 0.1 to 0.7, from left to right).

Therefore, we now compare the evolvability and mutational robustness of pioneers with that of those forming the majority. Because these are, in many cases, independent evolutionary trajectories, we recorded the line of descent for both groups and investigated the organisms at the beginning of this line and those at the end, when they became either pioneers or part of the majority. Consequently, we can compare how pioneers adapt their mutational robustness over the course of evolution with that of majority-forming organisms. We found that in low-mutation environments, pioneers experience an increase in the effect of mutations over the course of evolution (the mean Hamming distance caused by mutations increases; see Figure 6, black lines for ancestral pioneers and gray lines for adapted pioneers). In other words, they increase their evolvability by increasing the effects mutations have. (Some measurements show no significant difference, likely due to the small number of events in which either a pioneer found the highest peak or a majority was formed later. We base our generalizations on the data points that showed a statistically significant difference).

In environments with a higher mutation rate, pioneers slightly reduce the effect of mutations, increasing mutational robustness. In environments with higher mutation rates, we find that organisms start with stronger mutational effects and reduce these over the course of evolution (red lines in Figure 6). As such, they become more mutationally robust and can, as expected, remain at the higher peak, which, the pioneers, who needed higher evolvability, could not.

In other words, evolvability needs to be increased when mutation rates are low in order to cross the valley, but this happens rarely. As the mutation rate increases, it becomes harder for organisms with high evolvability and low mutational robustness to stay at the highest peak. Consequently, they become replaced by organisms that reduce their evolvability and instead increase their mutational robustness. The advantage of exploring the landscape due to heightened evolvability is replaced by the need for mutational robustness to stay at higher peaks as the mutation rate increases.

### 3.3. Competitive Advantage of Pioneers Depends on Their Mutational Robustness

To illustrate the relationship between evolvability and the ability to form a majority at the highest peak while competing against organisms at the lower peak with elevated mutational robustness, we performed the following experiment: by seeding the two-peak landscape with a single organism at the highest peak and 99 organisms at the lower peak, we can study the competitive advantage of this single pioneer. While this setup removes diversity in the population and the heterogeneity in the landscape occupation, it allows us to count how often the pioneer sweeps the population and how often it goes extinct.

Without further manipulation, one would expect the pioneer—having a fitness of 1.0—to easily outcompete the other organisms, which, in this experiment, all have the fitness of the lower peak set to 0.7, with the valley depth set to 0.1. We then artificially increase or decrease the mutational robustness (and thus the evolvability) of the starting organisms and perform competition experiments between all possible combinations of evolvable and robust organisms at both peaks (see Materials and Methods for details). Our previous findings suggest that an organism with high evolvability tends to find the highest peak first, while organisms with high mutational robustness remain at the secondary peak. This competition should significantly reduce the ability of a highly evolvable pioneer to form a majority. Conversely, if a mutationally robust organism is at the highest peak, it should have an elevated chance to outcompete more evolvable (less mutationally robust) organisms at the secondary peak.

**Figure 7:**
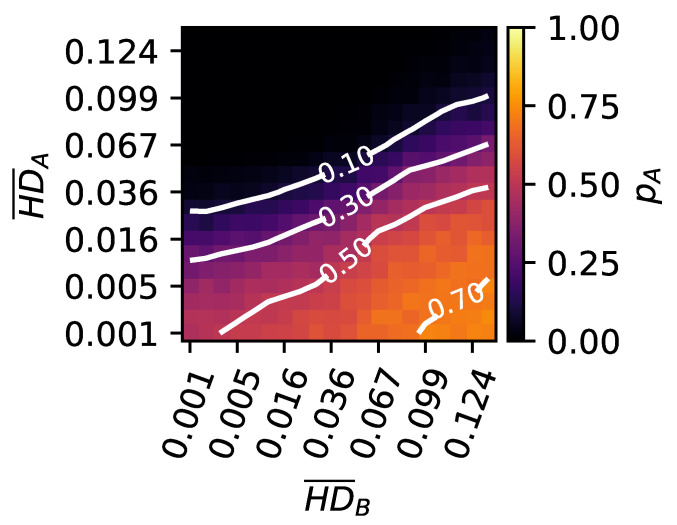
Heatmap showing the probability of a single organism at the highest peak, given different degrees of evolvability (${\bar{HD}}_{A}$) to outcompete 99 other organisms at the lower peak, also with different degrees of evolvability (${\bar{HD}}_{B}$). See the legend on the right about colors relating to probabilities. Isobars are shown for clarity as well. Here, 900 replicate experiments were used to determine the probability for the organism at the peak to win ($p_{A}$). The landscape had the fitness at the secondary peak set to 0.7, the valley at 0.1, and the mutation rate at μ=0.01.

We illustrate this principle in an environment with a high mutation rate (μ=0.01). As expected, the likelihood of the pioneer outcompeting the rest of the population depends on the evolvability (measured as the mean Hamming distance induced by mutations, $\bar{HD}$) of both competitors (see Figure 7). Highly evolvable organisms at the highest peak (high ${\bar{HD}}_{A}$) have less than a 10% chance to outcompete highly mutationally robust organisms at the lower peak (low ${\bar{HD}}_{B}$) (top left corner of Figure 7). Only highly mutationally robust organisms (low ${\bar{HD}}_{A}$) at the highest peak can outcompete organisms at the lower peak (winning more than 70%) when those are highly evolvable (high ${\bar{HD}}_{B}$) (see bottom right of Figure 7). The results, when testing different mutation rates, valley depths, or heights of the secondary peak, further support our hypothesis (see Supplementary Figure S1).

Note that this is a highly simplified environment, as the organisms at the highest peak have not evolved from those at the lower peak. Furthermore, the range of Hamming distances has been artificially inflated beyond what we have observed in other experiments. Therefore, this experiment serves as an illustration of the phenomenon rather than a quantitative representation of the evolutionary dynamics in the computational model.

### 3.4. Changes in Pleiotropy and Epistasis Convey Mutational Robustness and Evolvability

Now that we have shown how mutational effects can increase or decrease as an adaptation to different mutation rates or environmental conditions (valley height and height of the secondary peak), we focus on the changes in ϵ and π.

Given the previous experimental conditions, and the difference between pioneers and those forming a majority, we again observe the ancestors and those who become pioneers or form a majority.

**Figure 8:**
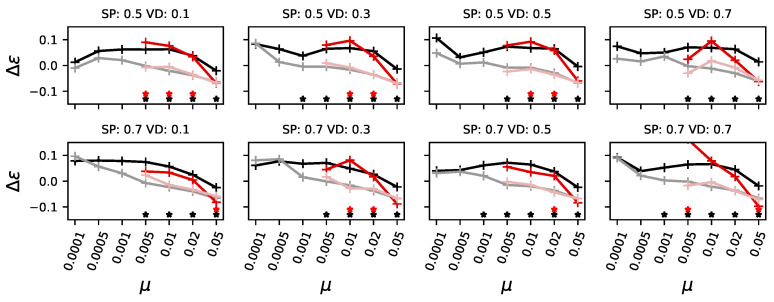
Epistasis of organisms at different experimental conditions. In black, the ancestors of pioneers who found the highest peak; the latter is shown in gray. In pale red, the organisms who form a majority, and in dark red, their ancestors. Stars indicate a significant (*p*-value of a Kolmogorov–Smirnov test less than 0.01) difference between ancestors and adapted organisms for pioneers in black and for those forming a majority in red. Each panel shows the mutation rate (μ) on the x-axis. Panels also vary the height of the secondary peak (SP, top row for SP=0.5 and bottom row SP=0.7, and the fitness at the deepest point of the valley (valley depth (VD), ranging from 0.1 to 0.7 from left to right).

**Figure 9:**
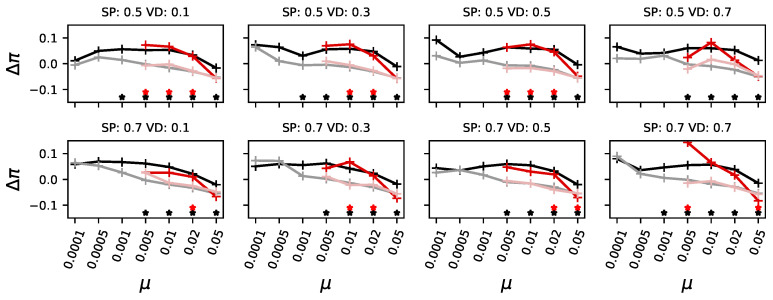
Pleiotropy of organisms at different experimental conditions. Axes, colors, and layout are identical to those in Figure 8.

We find that pioneers generally start with a higher level of ϵ and π (see Figure 8 and Figure 9, black lines being larger than 0.0), and over the course of evolution, reduce both significantly only if the mutation rate becomes large (same figures, gray lines). When the mutation rate is sufficiently high for the pioneers to become outcompeted by those who form a majority, we see the same kind of adaptation on these organisms (see Figure 8 and Figure 9, red and pale red lines). Interestingly, when mutation rates are extremely high (μ>=0.05), it appears that the difference between the ancestors and those who form the majority becomes smaller over the course of evolution—it is possible that this is an artifact of populations experiencing a mutational meltdown [31].

To support the notion that mutational robustness is conveyed by a reduction in pleiotropy and epistasis and that evolvability is conveyed by their increase, we generated random organisms and enriched the dataset with those who have a higher or lower ϵ and π. For this dataset of 60,000 samples, we also measured the mean Hamming distance of mutations and correlated that to ϵ and π.

**Figure 10:**
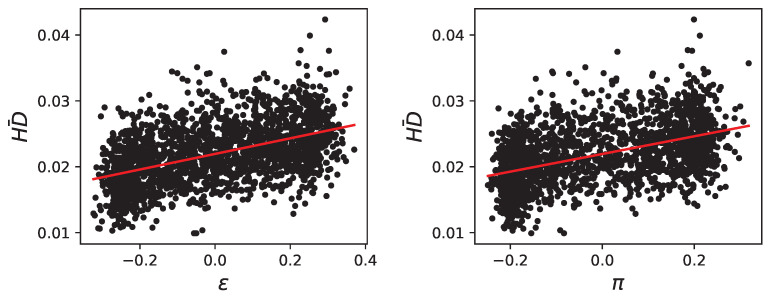
Scatter plot of the mean Hamming distance of mutants ($\bar{HD}$ y-axes) on randomly generated organisms enriched with high and low epistatic and pleiotropic samples and their epistasis (ϵ x-axis, left panel) and pleiotropy (π x-axis, right panel). A linear fit is shown in red (vanishing *p*-values for the fit). The dataset has 60,000 samples, only 2000 random points are shown for visibility reasons.

As expected, we observe a significant correlation between the mean Hamming distance of mutants and both epistasis (ϵ) and pleiotropy (π) (see Figure 10). We performed polynomial regression analysis using Ordinary Least Squares (OLS) to assess the relationship between these genetic interactions and mutational robustness. The results indicate that the correlations are highly significant, with *p*-values less than $1\times 10^{-16}$ for epistasis and pleiotropy. Additionally, the models exhibit strong explanatory power, achieving R-squared values of 0.957 for epistasis and 0.942 for pleiotropy. These findings underscore the robust association between increased epistasis and pleiotropy and the mean Hamming distance of mutants, reinforcing the role of these genetic interactions in shaping mutational robustness.

## 4. Discussion and Conclusions

In this study, we investigated the role of epistasis (ϵ) and pleiotropy (π) on evolvability and mutational robustness in a simple two-peak landscape. Organisms possess an indirect encoding, which means that they can evolve the degree in which their genes interact, and with that, ϵ and π can adapt to their environment and influence evolvability and robustness. We showed earlier that a reduction in both improves mutational robustness and allows mutants to stay at the higher peak under a higher mutation rate and thus evade the survival of the flattest fate.

This landscape is, of course, a simplified model, and a natural fitness landscape might deviate, but we still think that our findings can be generalized in principle. The key factors that varied were the mutation rate, the valley’s depth, and the height of the secondary peak where populations started to evolve. We measured the likelihood that the primary higher peak was found, and at which point, a majority population was formed, indicating that a stable population was established.

Interestingly, we made two observations that explain the role of evolvability and mutational robustness in more detail. First, pioneers, organisms who find the higher peak first, struggle to cross the valley at low mutation rates and, as expected, profit from an increase in evolvability. We found an increase in the mutational robustness of these pioneers in the high-mutation-rate regimes. The novel observation here is that this increase in evolvability is linked to an increase in ϵ and π. Similarly, in high-mutation regimes, the mutational robustness related to a decrease in ϵ and π improves in the case of crossing a valley, and not only in helping to stay at a fitness peak [18].

The second observation is about the inability of pioneers to form stable populations as the mutation rate increases. These pioneers go extinct and are replaced by organisms from the secondary peak, which, instead of becoming more evolvable, reduce their ϵ and π, and become more mutationally robust; they then conquer the primary higher peak. In low-mutation-rate environments, pioneers who increased their evolvability cross the valley and find the highest peak, even though this remains a rare event. However, in high-mutation-rate environments, mutational robustness becomes increasingly important. The actual establishment of a population at the higher peak is carried out by organisms that improve their mutational robustness by decreasing ϵ and π.

This observation, which is not always statistically significant due to the rarity of pioneers with increased evolvability, generally follows our intuitions regarding the valley’s depth or the secondary peak’s height. However, we did not vary the distance between the peaks, change the width of the path across the valley, or add additional noise or ruggedness to the very simple landscape; all factors could impose further constraints and might be investigated further.

Although our study is based on a computational model, an empirical validation of our findings would enhance their applicability to biological evolution. Investigating natural populations or conducting experimental evolution studies could test our hypothesis regarding evolvability, mutational robustness, and the roles of epistasis and pleiotropy. Specifically, we propose that early evolutionary stages benefit from increased evolvability, which can be facilitated by an increase in epistasis and pleiotropy. In contrast, organisms that have reached a fitness peak enhance their mutational robustness by reducing epistasis and pleiotropy. Consequently, we should expect to find that natural organisms follow the same trend.

We modeled asexual haploid organisms to maintain the focus on the evolutionary dynamics of valley crossing and the role of mutational robustness, evolvability, epistasis, and pleiotropy. Incorporating diploid genetics and recombination mechanisms, such as sexual reproduction, introduces substantial complexity due to factors like gene dominance, recessivity, and the intricate interactions between paired chromosomes. These complexities present significant challenges in accurately modeling evolutionary processes and would require addressing numerous unresolved questions, such as determining dominance relationships and managing the vast interaction possibilities between gene copies. Additionally, sexual reproduction and recombination can profoundly influence genetic diversity, fitness landscapes, and the mechanisms of valley crossing, potentially altering the outcomes observed in our asexual model. While exploring sexual reproduction and recombination is undoubtedly valuable and could provide deeper insights into evolutionary biology, it falls beyond the scope of our current study. Future research may extend our model to incorporate these mechanisms, allowing for a more comprehensive understanding of how sexual reproduction and genetic recombination affect epistasis, pleiotropy, and mutational robustness within evolutionary systems.

To gain a deeper understanding of fitness landscape dynamics, integrating topological analyses of genetic and phenotypic networks could provide valuable insights. Examining network properties such as modularity, connectivity, and clustering might reveal how specific configurations influence the adaptability and robustness of populations. While our current study does not explicitly explore these network characteristics, future extensions could test how variations in network topology affect evolutionary trajectories.

Furthermore, the trade-offs between evolvability and robustness elucidated in our study have significant implications for real-world biological scenarios. For instance, in microbial adaptation to antibiotics, bacteria must balance the ability to develop resistance (evolvability) with maintaining essential functions despite genetic changes (robustness). Our findings suggest that higher evolvability, facilitated by increased epistasis and pleiotropy, may enable rapid resistance development, but this comes at the cost of long-term stability, potentially making populations vulnerable once selective pressures change. This also raises the question about the difference between static and dynamically changing landscapes, which we look forward to studying in the future [32].

Beyond biological applications, our findings hold relevance for evolutionary computation. Often, systems that are optimized should be evolvable to improve the search. We suggest that increasing epistasis and pleiotropy might serve as a method to do so. At the same time, a genetic algorithm might not suffer from the long-term stability issues we identified, as the algorithm can stop once a solution is found. However, without such early stopping, robustness needs to be considered.

Lastly, the model of indirect encoding used here is rather simplistic, and more complex ones that include development, gene regulatory networks, or other factors have been proposed [33,34,35]. While we think that our observations should in general hold true in more complex models, they might have a more natural way of defining epistasis and pleiotropy.

## 5. Conclusions

In conclusion, our study explores the role of epistasis (ϵ) and pleiotropy (π) in balancing evolvability and mutational robustness during adaptive evolution, specifically regarding valley crossing. We observed that pioneers crossing fitness valleys benefit from increased evolvability through higher ϵ and π, particularly at low mutation rates, aiding in the discovery of superior fitness peaks. Conversely, at high mutation rates, populations enhance mutational robustness by decreasing ϵ and π, enabling them to establish and maintain stable populations on higher peaks despite the prevalence of deleterious mutations. These dynamics suggest that organisms may adaptively modulate their genetic interactions – increasing epistasis and pleiotropy to explore new adaptive peaks during early evolution and reducing them to maintain stability once a peak is reached. This adaptive behavior was studied in the limited context of a single valley crossing, raising the question of how this behavior generalizes to longer evolutionary adaptations in rugged fitness landscapes.

## Figures and Tables

**Table 1:** All experimentally varied and constant parameters of the computational model.

Parameter	Value or Range
population size	100 (constant)
number of generations	10,000 (constant)
structure	well mixed
reproduction mode	asexual, no recombination
replacement mode	the entire population is replaced
	at every generation
selection mechanism	roulette wheel
genome size	with N=20 we have 420 genomic
(*N* expression values and N×N interactions)	components defining the phenotype
phenotype size (number of traits *N*)	20
point mutation rate (μ)	varies: 0.0001, 0.001, and 0.01
primary peak height $W_{a}$	1.0 (constant)
secondary peak height $W_{b}$ or SP	varies: 0.5 or 0.7
valley height $W_{c}$ or VD	varies: 0.1, 0.3, 0.5, or 0.7
distance from primary to secondary peak	7 (Hamming distance)
distance from primary peak to valley	2 (Hamming distance)
independent replicates for the evolution experiment	
independent replicates for correlation plot	60,000
independent replicates for the	900
competition experiment	(for each of the 20×20 pairings)
independent replicates for competition	50
experiment (Supplementary Figure S1)	(for each of the 20×20 pairings)

## Data Availability

All data and code will be made available through GitHub upon acceptance of the manuscript for publication.

## Supplementary Materials

The following supporting figure can be downloaded at https://www.mdpi.com/article/10.3390/biology13121003/s1, Figure S1: Competitions between single organisms at the highest peak and the remaining population at the secondary peak, given different mutation rates, valley depths, secondary peak heights, and levels of mutational robustness.

## Abbreviations

The following abbreviations are used in this manuscript:

SoF	survival of the flattest
ϵ	epistasis
π	pleiotropy
μ	mutation rates
