# Supplementary material for: The Role of Pleiotropy and Epistasis on Evolvability and Robustness in a Two-Peak Fitness Landscape
Source: Biology (Basel). 2024 Dec 2;13(12):1003. doi: 10.3390/biology13121003 (PMC11727495; doi:10.3390/biology13121003)
Supplement: Supplementary file 1 [file biology-13-01003-s001.zip › biology-3256090-supplementary.pdf]

Supplementary Figure S1

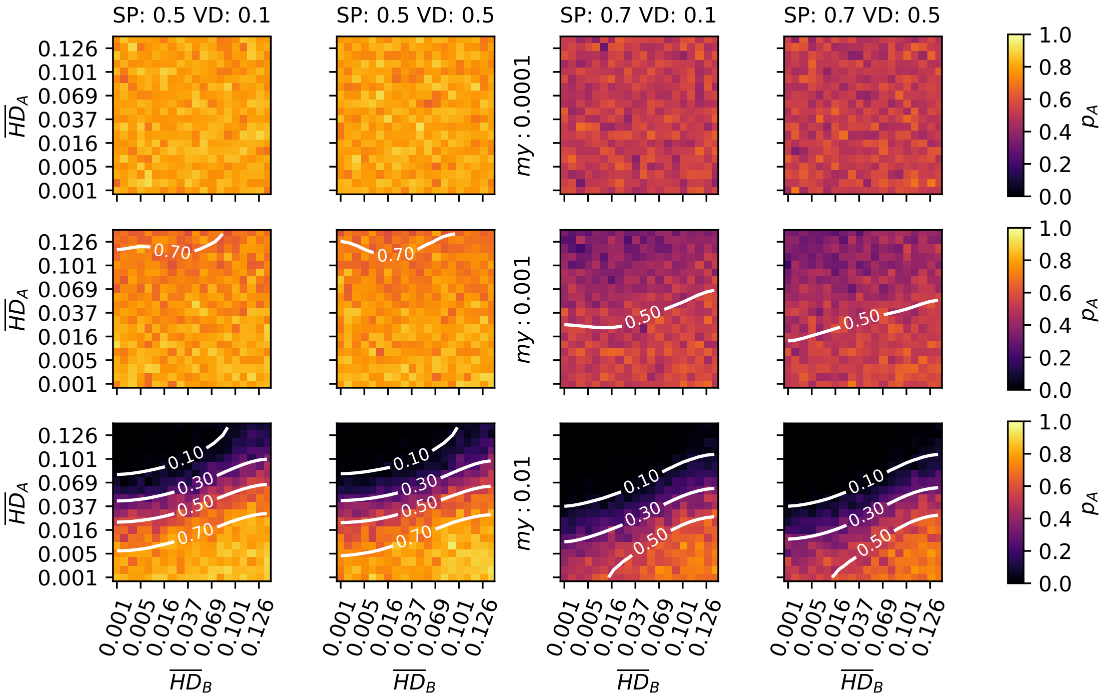

Figure S1: Competitions between single organisms at the highest peak and the remaining population at the secondary peak, given different mutation rates, valley depths, secondary peak heights, and levels of mutational robustness. The tested valley depths of 0.1 and 0.5 and the height of the secondary peak differ from column to column (see title), while each row shows results for different mutation rates (see label in the center of the figure). Each heatmap shows the probability of a single organism at the highest peak, given different degrees of evolvability ($\overline{HD}_A$, measured as the mean Hamming Distance between the organism and its possible mutants – $\mu = 0.01$ sampled using 10.000 mutants), to outcompete 99 other organisms at the lower peak, also with different degrees of evolvability ($\overline{HD}_B$). See the legend on the right about colors relating to probabilities. Here, 80 replicate experiments were used to determine the probability for the organism at the peak to win (p_A). Due to the reduced sample size, isobars were smoothed by applying a Gaussian filter ($\epsilon = 3.0$).
